# Racial Disparities in Emergency Department Utilization for Dental/Oral Health-Related Conditions in Maryland

**DOI:** 10.3389/fpubh.2017.00164

**Published:** 2017-07-18

**Authors:** Natalia I. Chalmers

**Affiliations:** ^1^Analytics and Publication, DentaQuest Institute, Columbia, MD, United States

**Keywords:** oral health, emergency service, hospital, healthcare disparities, cost of care, Maryland

## Abstract

**Objectives:**

Hospital emergency departments (EDs) are a place where many Americans seek treatment of dental conditions. Racial and ethnic minorities consistently have higher rates of ED utilization than whites for dental conditions. The reasons for these disparities and significant public health concerns are investigated less often. In this paper, we measure trends in racial disparities in ED discharges for dental conditions in Maryland from 2010 to 2013. To understand these disparities, we also describe differences between racial groups in age, gender, income, location, payer, comorbidities, and the availability of dental care.

**Methods:**

2010–2013 State Emergency Department Data for Maryland were used in the analysis. Rates per 100,000 of the population are calculated using information from census population estimates. Cost-to-charge ratios are used to estimate the costs of ED discharges. Dental/oral health-related conditions (DOHRC) are defined as discharge diagnoses of ICD-9-CM codes 520.0 through 529.9. Descriptive statistics and fixed effects logistic regression models with a rare event correction are used to analyze the data.

**Results:**

Blacks, especially females aged 25–34, have larger proportions of total ED discharges due to DOHRC, and higher population rates of DOHRC, than any other racial or ethnic group. In 2013, Blacks represented 30% of Maryland’s population and accounted for 52% of ED costs for DOHRC. Hispanics and those of other races have much lower rates of DOHRC discharges. The regression results show that the high proportion of DOHRC discharges among Blacks may be explained by the concentration of Blacks in low-income central cities with less access to dental care.

**Conclusion:**

There are significant racial disparities in the ED utilization for DOHRC in Maryland. These disparities reflect the lack of access to dental care due to both cost and geographic limitations. This results in high healthcare costs and ineffective solutions for patients. Addressing oral health disparities will require policy solutions that are targeted to the populations most at need, and action plans that combine community and state level efforts.

## Introduction

Americans increasingly utilize hospital emergency departments (EDs) for the treatment of dental conditions (1, 2). These visits are primarily palliative and underlying problems are rarely satisfactorily treated potentially resulting in further pain, worsening oral health, and additional cost for patients and families (3). These visits are frequently caused by a lack of access to affordable dental care (4–6).

Racial disparities in the utilization of EDs for dental conditions are a consistent finding in research about this public health concern. Evidence from Wisconsin, New Jersey, and Maryland demonstrates that Black Americans, especially those with no or public insurance and of low socioeconomic status, have higher rates of ED visits than whites (7–10). The most commonly cited reasons for these high rates include poor overall and oral health combined with a lack of access to dental insurance and care (7–9, 11–13). However, little explanatory work has been done in this area. Evidence regarding other racial groups is much more limited. Some evidence suggests that Hispanics have higher rates of ED visits for dental conditions due to a lack of access to dental care and lower average health literacy (14). However, there is a well-known “Hispanic Paradox” in which Hispanics have better overall and oral health than might be expected, given their average socioeconomic status (11, 15).

In this paper, we assess racial disparities in ED discharges for dental conditions in Maryland from 2010–2013. We chose to focus on Maryland for several reasons. First, recent public and policy attention in Maryland has been focused on reducing disparities in access to oral health care (16). Second, ED discharges for dental conditions in Maryland are well understood and mirror national trends (9, 14, 17). As occurs nationally, EDs in Maryland are not capable of providing definitive treatment of dental conditions. Therefore, many patients either see a dentist to complete treatment or return to the ED for further palliative care and their condition is left unresolved (9, 17). The demographics of Maryland are somewhat different from national averages. In particular, Maryland is more racially diverse, has higher average income, and is slightly more urban than national averages (18). Finally, despite the wealth of research and programs in Maryland, there has been no comprehensive research documenting differences in ED discharges for dental conditions across racial groups.

## Materials and Methods

### Data

The data used in this paper come from the 2010 to 2013 State Emergency Department Databases (SEDD) of the Healthcare Cost and Utilization Project (HCUP) for Maryland. A dataset that pooled discharges from the 2010 through 2013 datasets was created to facilitate comparisons over time. These data and analysis are representative of the total population of ED discharges over this period. When reported, rates per 100,000 of the population were calculated using information from county level population estimates (18). Information on the number of dentists and ratio of dentists to population comes from the Area Resources Health Files (19). Cost-to-charge ratios from HCUPs were used to estimate the costs of ED discharges (20). These ratios reflect inpatient costs but have been used in prior research on ED discharges (2) and are the best available tool for estimating the actual costs of ED visits.

### Variables

Dental/oral health-related conditions (DOHRC) are defined as diagnoses of ICD-9-CM codes 520.0 through 529.9. Variables indicating preventability and severity of DOHRC were created using a schema that classifies ICD-9-CM dental diagnosis codes based on the severity of the condition and the likelihood that the conditions were preventable through normal dental care (21). In order to ensure an adequate sample size to analyze differences between racial groups, we collapsed race into four categories: Whites, Blacks, Hispanics, and other races. Comorbidities are defined using the expanded Elixhauser comorbidities index (22). Other variables in this analysis include age, gender, income, location, payer, and the availability of dental care.

### Methods

When conducting descriptive analysis, Chi-square tests are used to determine if there are differences between expected and observed distributions of the independent variables and the dependent variables. The statistical significance of individual cells of data is determined by the contribution of that cell to the overall Chi-square using a *p* value cutoff of <0.05. Results of these descriptive statistical tests are presented in Appendix S1 in Supplementary Material.

A fixed effects logistic regression model that contrasts DOHRC discharges with other discharges is estimated. These models “fix” or control for the effect of time, producing estimates that show year over year averages. The relatively rare nature of DOHRC discharges in the full sample necessitates the use of a rare event correction to ensure accurate predictions. The rare event correction is performed by randomly selecting a 5% subsample of non-DOHRC discharges to create a balanced sample and then a correction for the resulting sample selection bias (23).

Two models are estimated. The first estimates the effect of race on the odds of being discharged with a DOHRC relative to any other condition. The second model adds variables that may mediate the relationship between race and odds of having a DOHRC discharge, as identified by the descriptive analysis. These variables include a gender by age interaction, the rate of dentist per 100,000 of population in the county, location of residence, and median household income in the county of residence.

## Results

Dental/oral health-related conditions discharges are not evenly distributed by race (Table 1). For all years, between 3.0 and 3.1% of all ED discharges among Blacks are due to DOHRC, the highest among racial groups and higher than the population average of 2.7–2.8%. In comparison, only 1.3–1.5% of ED discharges among Hispanics and those of other races are due to DOHRC (see Appendix S2 in Supplementary Material for information on all ED discharges). Across all years, Blacks also have by far the highest population rates of DOHRC discharges and the proportion of DOHRC discharges among this racial group increases from 48% in 2010 to 52% by 2013. Whites have the next highest population rate of DOHRC discharges, but those rates decline from 2010 to 2013. Hispanics and those of other races have low, but increasing, rates of DOHRC.

**Table 1:** Maryland emergency department (ED) discharges for dental/oral health-related conditions (DOHRC) by race using 2010–2013 State Emergency Department Data (SEDD).

	Count of DOHRC	Rate per 100,000 of population	%	% of total ED discharges	Average cost ($)	Total cost ($)	Count of DOHRC	Rate per 100,000 of population	%	% of total ED discharges	Average cost ($)	Total cost ($)
		
	2010						2011					
White	26,641	841.27	48	2.8	222	5,246,897	27,276	861.04	47	2.7%	248	5,614,868
Black	26,475 ↑	1,570.50	48	3.0	255	6,041,558	27,628 ↑	1,621.94	48	3.0%	279	6,668,503
Hispanic	1,134 ↓	238.57	2	1.4	263	266,266	1,187 ↓	239.50	2	1.3%	263	277,131
Other	1,315 ↓	285.72	2	1.5	247	283,132	1,368 ↓	286.78	2	1.5%	303	344,071
Total	55,565	959.99		2.8	239.05	11,837,853	57,459	983.24		2.7%	264.53	12,904,573

	**2012**						**2013**					
	
White	26,648 ↓	841.68	45	2.6	280	6,269,330	23,166 ↓	733.14	43	2.5	338	6,709,003
Black	29,973 ↑	1,745.11	50	3.0	317	8,345,708	27,793 ↑	1,602.58	52	3.1	342	8,259,798
Hispanic	1,563 ↓	303.50	3	1.5	277	376,909	1,452 ↓	271.24	3	1.3	302	365,831
Other	1,604 ↓	325.21	3	1.4	334	433,411	1,450 ↓	284.68	3	1.4	333	399,606
Total	59,788	1,014.76		2.7	300.38	15,425,358	53,861	906.94		2.7	333.98	15,734,238

*↑ indicates Chi-square significantly higher than expected and ↓ indicates significantly lower than expected. DOHRC are defined as diagnoses of ICD-9-CM codes 520.0 through 529.9. Estimates from Maryland SEDD, 2013, Agency for Healthcare Research and Quality (AHRQ). Costs calculated using cost-to-charge ratio files for the state inpatient databases, 2013, AHRQ*.

**Figure 1:**
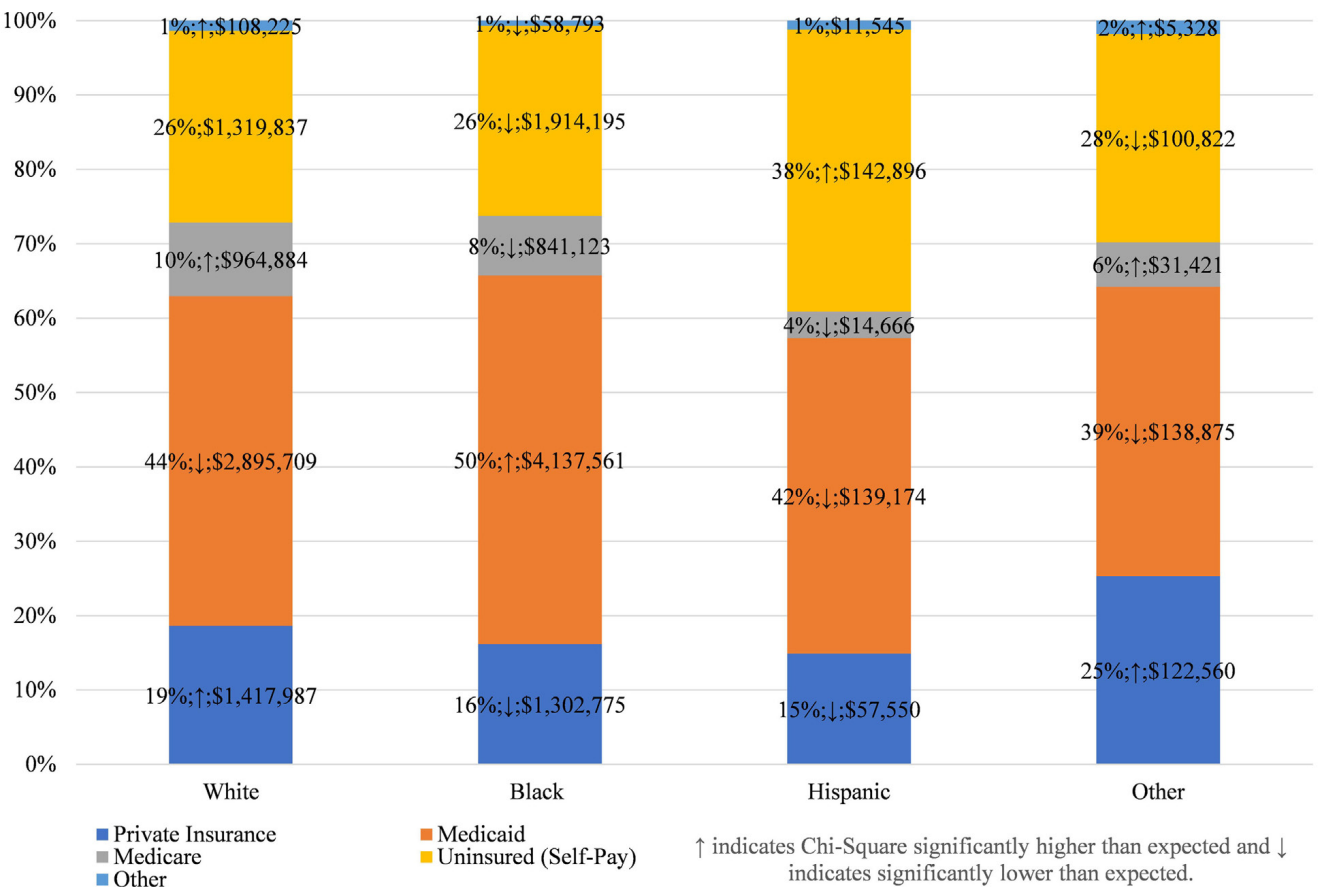
Expected payers of dental/oral health related conditions discharges, by race, in Maryland 2013.

This concentration of inappropriate and ineffective ED treatment among a single racial group has implications beyond the disservice to the patients involved. In total, $15.7 million was spent in Maryland in 2013 treating dental conditions in the ED, an increase of $4 million from 2010, adjusted for inflation (Table 1). On average, 51% of the total cost was spent on DOHRC discharges among Blacks, who represent about 30% of Maryland’s population. Moreover, because half of all Blacks discharged for DOHRC are enrolled in Medicaid in 2013, these discharges place a burden on taxpayers (Figure 1).

This payer mix is not, however, unique to Blacks. Among all racial groups, Medicaid is the largest payer of costs for ED DOHRC; followed by the uninsured themselves (Figure 1; see Appendix S3 in Supplementary Material for all years). Private insurance is more likely to pay for DOHRC discharges among Whites and other races than expected, while the Hispanic DOHRC patients are more likely to be uninsured.

**Figure 2:**
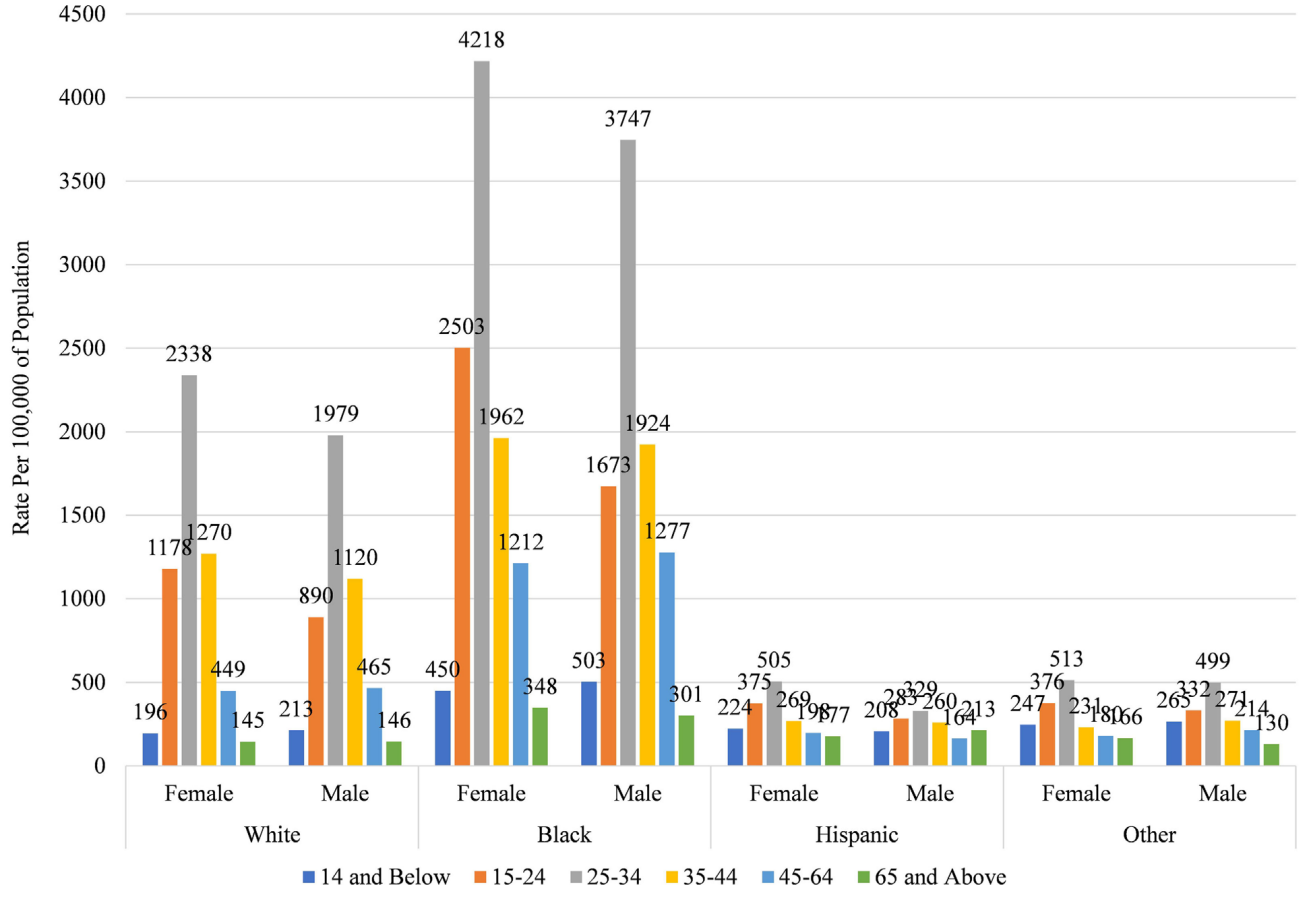
Rate of dental/oral health related conditions discharges by race, gender, and age in Maryland 2013.

Among all racial groups, females and those aged 25–34 have the highest rates of discharges for DOHRC (Figure 2 for 2013; Appendix S4 in Supplementary Material for all years). Within each gender and age category, Blacks consistently have the highest rates, while Hispanics and other races have the lowest rates. Black females aged 25–34 have a rate of 4,218 per 100,000, while Black males have a rate of 3,746. No other rate across the race, gender, and age distribution exceeds 2,500. Also of note is that rates for Whites and Blacks follow a much more pronounced distribution, with large peaks at 25–35, whereas those for Hispanics and other races are more evenly distributed across the age distribution.

**Figure 3:**
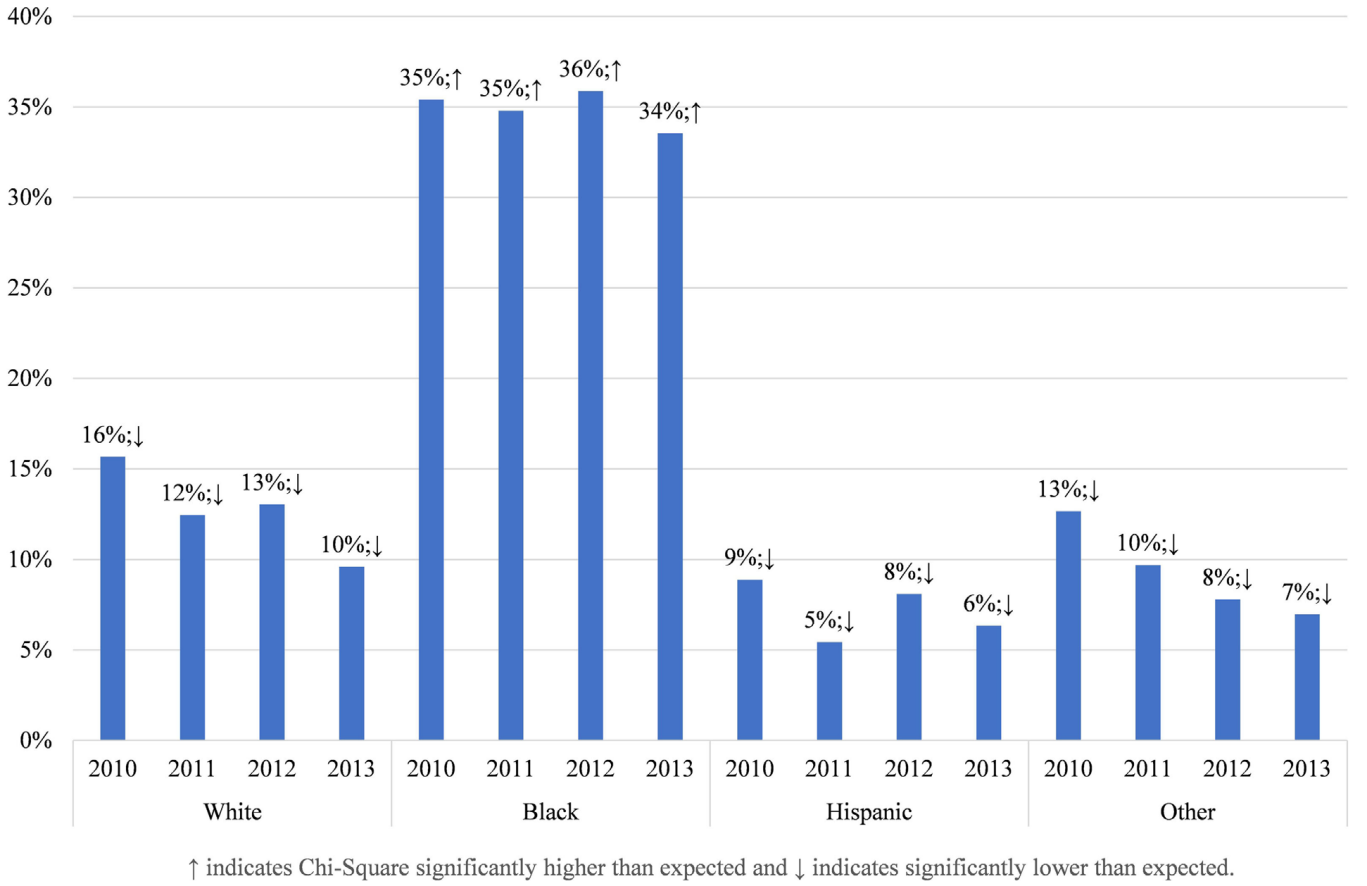
Proportion living in zip codes with median household incomes in bottom quartile, by race, in Maryland 2013.

**Figure 4:**
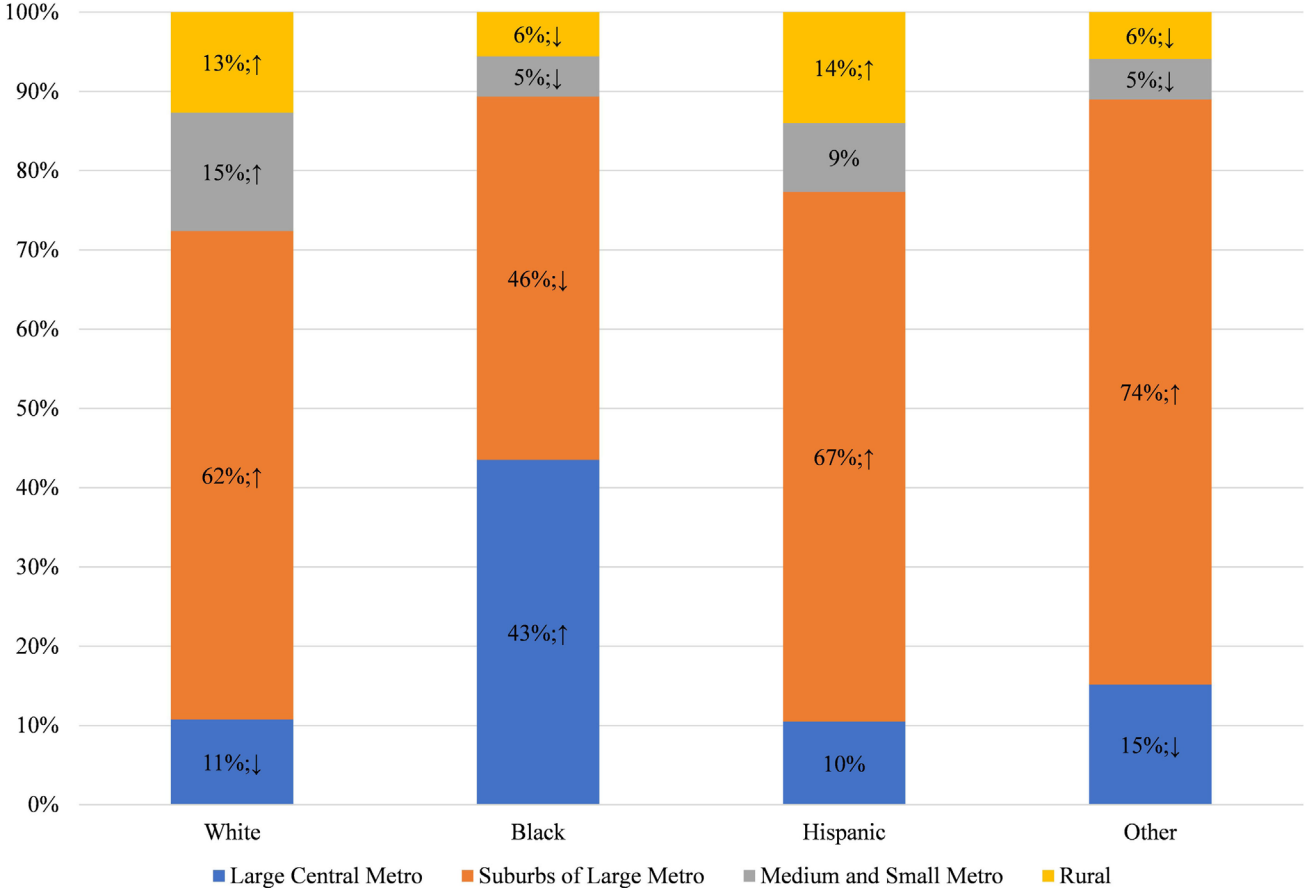
Location of residence of dental/oral health related conditions discharges, by race, in Maryland 2013.

To understand why these racial disparities in DOHRC discharges exist, we separate median household income of the zip code (Figure 3) and the location of residence (Figure 4) of the discharged patients by race. Blacks discharged with a DOHRC are disproportionately more likely to live in zip codes in the bottom income quartile. Across all years, about 35% of all Blacks discharged with a DOHRC live in zip codes where the median household income is less than $39,000 per year (Figure 3). In comparison, 16% or less of the other racial groups discharged with any condition live in low-income zip codes and the percentages for those groups decline over time. Hispanics and those of other races are the least likely to live in poor zip codes. Those discharged with a DOHRC, regardless of racial group, are more likely to live in zip codes with a median household income in the bottom quartile compared to those discharged with a medical condition (Appendix S5 in Supplementary Material). For example, 27% of Blacks discharged with a medical condition live in poor zip codes in 2013, compared to the 34% discharged with a DOHRC. Blacks discharged with a DOHRC are also more likely to live in a large central metro area (43%, Figure 4), relative to Blacks discharged with a medical condition (36%, Appendix S6 in Supplementary Material) or others. Two-thirds or more of Whites, Hispanics, and other races discharged with any condition live in the generally wealthier suburbs.

**Figure 5:**
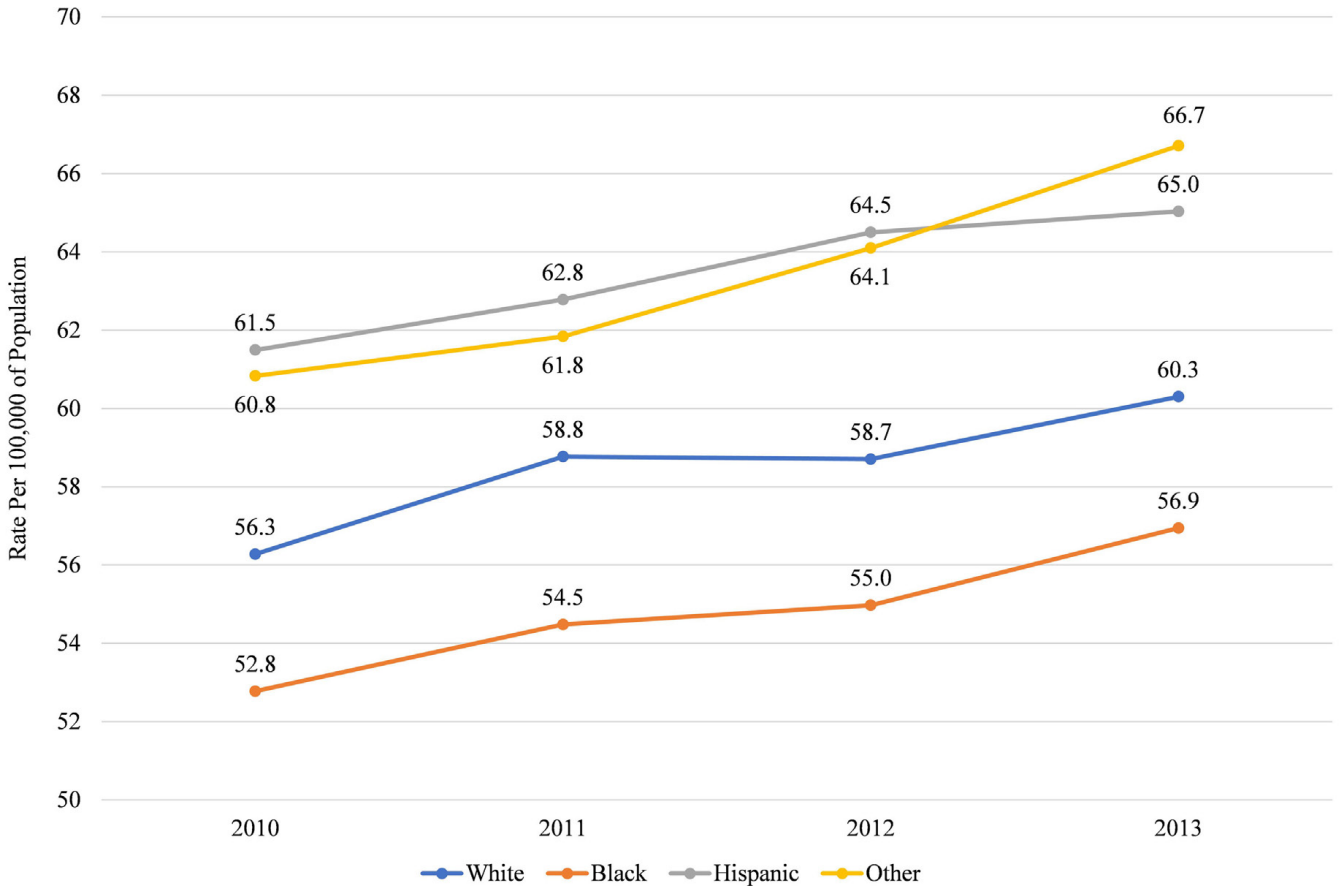
Median rate of dentists per 100,000 of population in county, by race, in Maryland 2010–2013.

**Figure 6:**
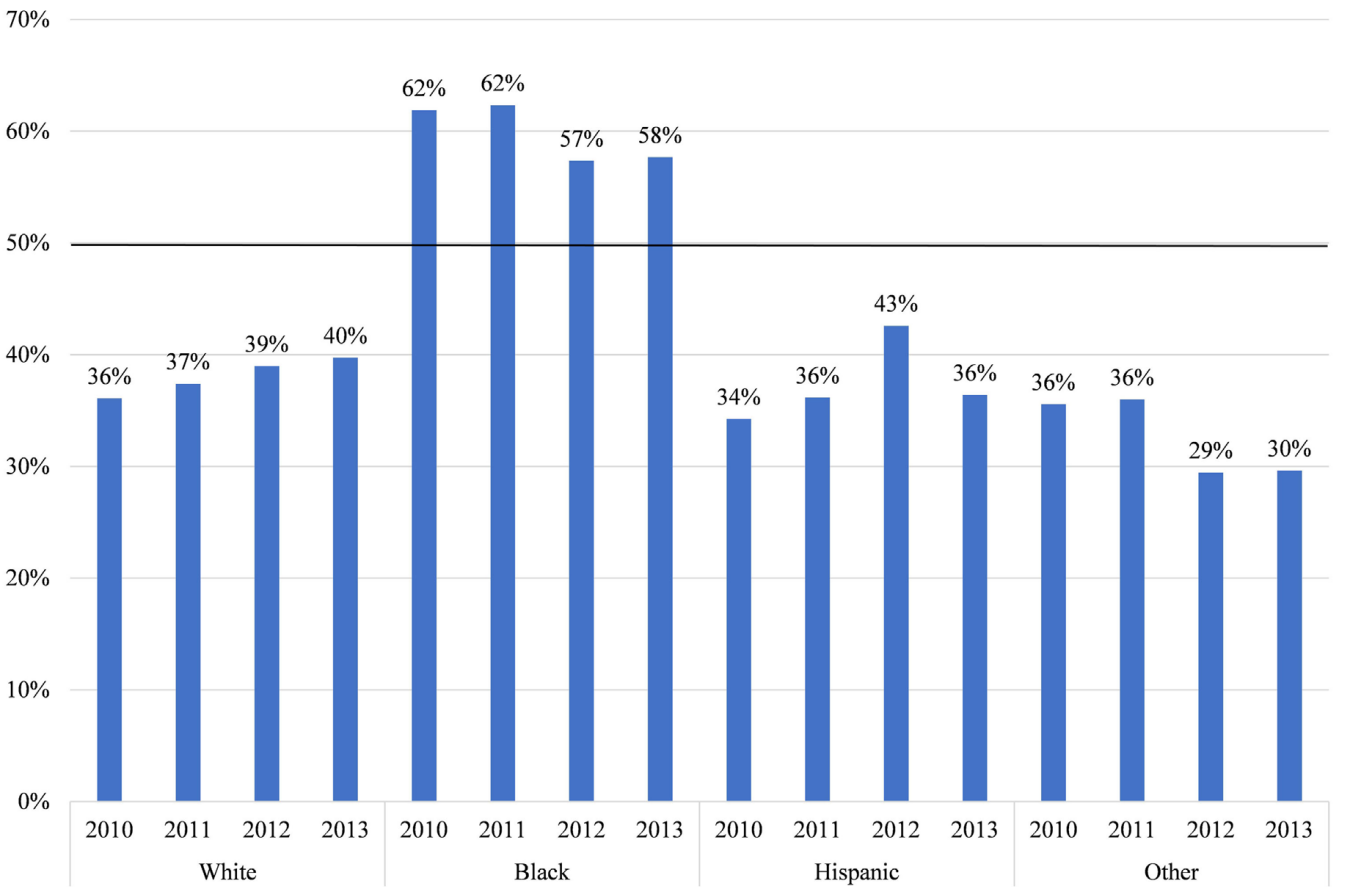
Proportion living in counties with below median rate of dentists per 100,000, by race, in Maryland 2010–2013.

This concentration of Blacks into low income, central city areas in Maryland is significant because Blacks are more likely to live in areas that have relatively few dentists per capita (Figure 5) and more than half live in counties that are below the median rate for dentists in the population (Figure 6). This finding is consistent with prior research that those living in low-income areas and those with low incomes are more likely to report that their mouth and teeth are in poor condition (24). It is also consistent with prior research that those living in low-income areas and those with low incomes are more likely to delay going to the dentist due to costs or difficulty finding a dentist (13, 24). Thus, Blacks may go the ED for dental care because they do not have access to the dentists, either because of geographic restriction or because of cost; and because EDs are relatively accessible and will treat patients with emergent conditions regardless of ability to pay. Comparatively, Hispanics and those of other races live in counties with more dentists per population (Figure 5) and less than half live in counties below the median rate of dentists (Figure 6). Thus, the low rate of ED discharges for these racial groups may be due to better access to dental care for these populations.

Racial disparities in ED discharges for DOHRC do not appear to be associated with differences in comorbidities (Appendix S7 in Supplementary Material). Regardless of race and ethnicity, those discharged with a DOHRC are less likely to have a chronic comorbidity that would complicate treatment than those discharged with a medical condition, with 22% or less having one or more. In addition, there are no differences in the preventability or the severity of dental conditions that are diagnosed in the ED that would explain the differences in rates seen across racial groups (Appendix S8 in Supplementary Material).

To test our hypothesis that disparities in ED discharges by race are due to residential segregation into high- and low-income areas with differential access to dentists, we estimate two fixed effects logistic regression models that estimate the odds of being discharged with a DOHRC relative to any other condition (Table 2). The first model, which includes only race, is reflective of the descriptive findings, Blacks are 20% more likely than Whites to be discharged with a DOHRC, relative to being discharged for a medical condition. On the other hand, Hispanics and those of other races are 40% less likely than Whites to be discharged for a DOHRC.

**Table 2:** Fixed effects logistic regression model estimating dental/oral health-related condition (DOHRC) discharges in Maryland using State Emergency Department Data (SEDD) 2010–2013.

	Model 1: race only			Model 2: race + potential mediators		
	DOHRC discharge (reference: all other discharges)			DOHRC discharge (reference: all other discharges)		

Independent variable	Odds ratio	SE	Pred. prob. of a DOHRC Diag.	Odds ratio	SE	Pred. prob. of a DOHRC diag.
Race						
(Reference: white)	–	–	0.25	–	–	0.27
Black	1.206*	0.006	0.29	1.002	0.006	0.27
Hispanic	0.600*	0.009	0.17	0.594*	0.010	0.18
Other race	0.594*	0.009	0.17	0.679*	0.011	0.20
Gender × age interaction						
(Reference: males 14 and below)	–	–	–	–	–	0.12
Males 15–24	–	–	–	3.504*	0.055	0.32
Males 25–34	–	–	–	6.036*	0.089	0.45
Males 35–44	–	–	–	3.731*	0.059	0.34
Males 45–64	–	–	–	2.151*	0.033	0.23
Males 65 and above	–	–	–	0.666*	0.017	0.08
Females 14 and below	–	–	–	1.102*	0.020	0.13
Females 15–24	–	–	–	0.846*	0.019	0.31
Females 25–34	–	–	–	0.725*	0.015	0.39
Females 35–44	–	–	–	0.721*	0.016	0.29
Females 45–64	–	–	–	0.762*	0.017	0.20
Females 65 and above	–	–	–	0.783*	0.027	0.07
Rates dentists per county population	–	–	–	0.997*	0.000	–
Location						
(Reference: large central metro)	–	–	–	–	–	0.27
Suburbs of large metro	–	–	–	0.965*	0.009	0.27
Medium and small metro	–	–	–	0.816*	0.010	0.24
Rural	–	–	–	0.871*	0.011	0.25
Household income quartile						
(Reference: $64,000+)	–	–	–	–	–	0.31
Less than $39,000 per year	–	–	–	1.690*	0.018	0.31
$39,000–$47,999 per year	–	–	–	1.634*	0.016	0.27
$48,000–$63,999 per year	–	–	–	1.331*	0.009	0.22
Year						
(Reference: 2013)	–	–	0.26	–	–	0.27
2010	1.077	0.008	0.27	0.996	0.008	0.26
2011	1.008	0.007	0.26	0.949*	0.007	0.26
2012	1.015	0.007	0.26	0.968*	0.007	0.27
Constant	0.327*	0.002	0.26	0.137*	0.003	0.26
Number of observations	869,741			869,741		
Model Chi-square test	5,153.62*	(6 *df*)		68,264.95*	(24 *df*)	
Goodness of fit Chi-square test	137.10*	(9 *df*)		22,812.52*	(9,492 *df*)	
Observations correctly classified	73.94%			73.8%		

*p < 0.001.

*DOHRC are defined as diagnoses of ICD-9-CM codes 520.0 through 529.9. Estimates from Maryland SEDD, 2010 through 2013, Agency for Healthcare Research and Quality (AHRQ). Rare event correction used, which uses the full sample of DOHRC discharges and a 5% random subsample of all other discharges*.

The second model in Table 2 adds variables that may mediate the relationship between race and odds of having a DOHRC discharge, as identified by the descriptive analysis. These variables include a gender by age interaction, the rate of dentist per 100,000 of population in the county, location of residence, and median household income of the county of residence. Those who live in counties with a greater density of dentists are less likely to visit the ED for a DOHRC. Those who live in a large central metro are more likely and those with high incomes are less likely to visit the ED for DOHRC, relative to visiting for a medical reason. Finally, there is a significant gender by age interaction, although once the other variables are accounted for, males are slightly more likely than females to visit the ED for a DOHRC.

When these variables are added to the model, the variable for Blacks becomes non-significant. This indicates that the difference between Blacks and Whites in the proportion of ED discharges that are due to DOHRC is caused by the relative disadvantage of Blacks in terms of income, location, and access to dental care. On the other hand, the variables for Hispanics and those of other races continue to be significant and with no or small reductions in magnitude. Therefore, the lower proportion of DOHRC for these groups is not associated with their relatively privileged position on the variables in the model.

## Discussion

In this paper, we identified significant racial disparities in ED discharges for DOHRC in Maryland between 2010 and 2013, with Blacks having a larger proportion of total ED discharges and higher population rates than any other group. Black females between the ages of 25–34 have by far the highest rates in the population. This disparity is not strongly related to worse overall health or different patterns of diagnoses in this population. Instead, as established in our regression models, it is associated with a lack of access to dental care due to cost and availability because of a concentration of this population in low-income and central city areas. Our findings are consistent with previous research that a higher level of ED utilization for dental conditions among Blacks is due to a lack of access to dental care. Our findings also support research documenting the better overall and oral health of Hispanics (11, 15), which in our analysis, cannot be explained by differences in access to dental care.

While the analysis presented here provides systematic evidence of racial disparities in ED utilization for dental conditions, there are obvious limitations. Because administrative data are used in the analysis, there is relatively little information on the patient themselves. Better information on the patient overall oral health status, dental insurance coverage, and their values related to oral health would provide more complete explanations of the reason and solutions for these racial disparities.

The ED is not the place for treatment of dental conditions for anyone. Costs are high and the care provided is palliative at best. The disproportionately high level of DOHRC discharges for Blacks in Maryland create high costs for Medicaid and ineffective solutions for patients. The disparities revealed in this paper highlight the importance of solutions targeted to the populations most at need. Any solution is going to need to combine community and government action to deal with lack of access to dental care due to location and cost. The proposed restoration of an adult dental care benefit for Maryland residents would allow Medicaid beneficiaries to seek dental care in more appropriate, and cost effective, settings (25, 26). Promising community interventions to divert patients from the ED to dentists that can deliver acute dental care have been successfully used to reduce ED visits for dental conditions in rural Maryland (27).

## Author Contributions

NC developed the concept and design of the study; NC contributed to the acquisition of data, analysis, and interpretation of data; NC made contribution to drafting and revising the article and the final approval of the version to be submitted.

## Conflict of Interest Statement

The author declares that the research was conducted in the absence of any commercial or financial relationships that could be construed as a potential conflict of interest.
