# Supplementary material for: Racial Disparities in Emergency Department Utilization for Dental/Oral Health-Related Conditions in Maryland
Source: Front Public Health. 2017 Jul 18;5:164. doi: 10.3389/fpubh.2017.00164 (PMC5515044; doi:10.3389/fpubh.2017.00164)
Supplement: Supplementary file 1 [file Data_Sheet_1.PDF]

Appendix 1: Statistical Tests from Tables and Figures

Table or Figure	Year	Statistical Test Type	Statistical Test Value	Degrees of Freedom	Significance
Table 1	2010	Chi-Square	1300	6	p<.001
Table 1	2011	Chi-Square	1500	6	p<.001
Table 1	2012	Chi-Square	2100	6	p<.001
Table 1	2013	Chi-Square	2100	6	p<.001
Figure 1	2013	Chi-Square	464.58	12	p<.001
Figure 3	2010	Chi-Square	3400	9	p<.001
Figure 3	2011	Chi-Square	4500	9	p<.001
Figure 3	2012	Chi-Square	4900	9	p<.001
Figure 3	2013	Chi-Square	5100	9	p<.001
Figure 4	2013	Chi-Square	8000	9	p<.001
