# Supplementary material for: Racial Disparities in Emergency Department Utilization for Dental/Oral Health-Related Conditions in Maryland
Source: Front Public Health. 2017 Jul 18;5:164. doi: 10.3389/fpubh.2017.00164 (PMC5515044; doi:10.3389/fpubh.2017.00164)
Supplement: Supplementary file 2 [file Data_Sheet_2.PDF]

Appendix 2: Maryland Emergency Department Discharges for Dental/Oral Health Related Conditions by Race using 2010-2013 SEDD

2010

	All Discharges					Dental Conditions					
	Count	Rate Per 100,000	%	Average Cost	Total Cost	Count	Rate Per 100,000	%	% of Total Discharges	Average Cost	Total Cost
White	948,443	29,950.03	48%	$486	$426,000,000	26,641	841.27	48%	2.8%	$222	$5,246,897
Black	869,644	51,587.38	44%	$484	$375,000,000	26,475	1,570.50	48%	3.0%	$255	$6,041,558
Hispanic	78,888	16,596.30	4%	$451	$32,900,000	1,134	238.57	2%	1.4%	$263	$266,266
Other	88,440	19,215.81	4%	$452	$36,400,000	1,315	285.72	2%	1.5%	$247	$283,132
Total	1,985,415	34,301.66		$482.27	$870,300,000	55,565	959.99		2.8%	$239.05	$11,837,853

2011

	All Discharges					Dental Conditions					
	Count	Rate Per 100,000	%	Average Cost	Total Cost	Count	Rate Per 100,000	%	% of Total Discharges	Average Cost	Total Cost
White	1,009,050	31,853.27	47%	$540	$492,000,000	27,276	861.04	47%	2.7%	$248	$5,614,868
Black	932,113	54,721.02	44%	$527	$432,000,000	27,628	1,621.94	48%	3.0%	$279	$6,668,503
Hispanic	91,146	18,390.22	4%	$467	$38,900,000	1,187	239.50	2%	1.3%	$263	$277,131
Other	92,743	19,442.45	4%	$492	$40,900,000	1,368	286.78	2%	1.5%	$303	$344,071
Total	2,125,052	36,364.01		$529.26	$1,003,800,000	57,459	983.24		2.7%	$264.53	$12,904,573

2012

	All Discharges					Dental Conditions					
	Count	Rate Per 100,000	%	Average Cost	Total Cost	Count	Rate Per 100,000	%	% of Total Discharges	Average Cost	Total Cost
White	1,018,137	32,157.94	46%	$658	$607,000,000	26,648	841.68	45%	2.6%	$280	$6,269,330
Black	986,566	57,440.44	44%	$632	$547,000,000	29,973	1,745.11	50%	3.0%	$317	$8,345,708
Hispanic	104,728	20,335.65	5%	$538	$50,200,000	1,563	303.50	3%	1.5%	$277	$376,909
Other	114,358	23,185.81	5%	$595	$58,600,000	1,604	325.21	3%	1.4%	$334	$433,411
Total	2,223,789	37,743.67		$638.08	$1,262,800,000	59,788	1,014.76		2.7%	$300.38	$15,425,358

2013

	All Discharges					Dental Conditions					
	Count	Rate Per 100,000	%	Average Cost	Total Cost	Count	Rate Per 100,000	%	% of Total Discharges	Average Cost	Total Cost
White	916,215	28,995.85	45%	$715	$584,000,000	23,166	733.14	43%	2.5%	$338	$6,709,003
Black	899,734	51,879.97	44%	$678	$528,000,000	27,793	1,602.58	52%	3.1%	$342	$8,259,798
Hispanic	108,856	20,334.79	5%	$576	$57,000,000	1,452	271.24	3%	1.3%	$302	$365,831
Other	104,201	20,457.92	5%	$642	$57,100,000	1,450	284.68	3%	1.4%	$333	$399,606
Total	2,029,006	34,165.61		$686.80	$1,226,100,000	53,861	906.94		2.7%	$333.98	$15,734,238

Dental/Oral Health Related Conditions are defined as diagnoses of ICD-9-CM codes 520.0 through 529.9. Estimates from Maryland State Emergency Department Data (SEDD), 2010-2013, Agency for Healthcare Research and Quality (AHRQ). Costs calculated using Cost-to-Charge Ratio Files for the State Inpatient Databases, 2010-2013, Agency for Healthcare Research and Quality (AHRQ).
