# Supplementary material for: Racial Disparities in Emergency Department Utilization for Dental/Oral Health-Related Conditions in Maryland
Source: Front Public Health. 2017 Jul 18;5:164. doi: 10.3389/fpubh.2017.00164 (PMC5515044; doi:10.3389/fpubh.2017.00164)
Supplement: Supplementary file 3 [file Data_Sheet_3.PDF]

Appendix 3: Payers of Charges for Maryland Emergency Department Discharges for Dental/Oral Health Related Conditions using 2010 SEDD

	2010															
	White				Black				Hispanics				Other			
Payer	Count	%	Average Cost	Total Cost	Count	%	Average Cost	Total Cost	Count	%	Average Cost	Total Cost	Count	%	Average Cost	Total Cost
Private Insurance	4,855	18.3%	$278	$1,220,971	4,791	18.2%	$254	$1,039,536	180	16.6% ↓	$258	$40,964	325	24.8% ↑	$279	$80,598
Medicaid	10,516	39.6% ↓	$201	$1,878,832	11,467	43.6% ↑	$255	$2,678,137	434	40.1%	$238	$90,090	443	33.8% ↓	$236	$90,682
Medicare	1,961	7.4%	$325	$581,736	1,909	7.3%	$300	$528,565	41	3.8% ↓	$253	$9,615	76	5.8% ↓	$301	$20,175
Uninsured (Self-Pay)	8,843	33.3% ↑	$189	$1,461,399	7,954	30.3% ↓	$242	$1,701,995	413	38.1% ↑	$293	$108,357	451	34.4% ↑	$226	$87,469
Other	371	1.4% ↑	$243	$83,262	172	0.7% ↓	$322	$48,991	15	1.4%	$222	$3,326	15	1.1%	$239	$3,342
Total:	26,546	100.0%	$222	$5,246,897	26,293	100.0%	$255	$6,041,558	1,083	100.0%	$263	$266,266	1,310	100.0%	$247	$283,132

	2011															
	White				Black				Hispanics				Other			
Payer	Count	%	Average Cost	Total Cost	Count	%	Average Cost	Total Cost	Count	%	Average Cost	Total Cost	Count	%	Average Cost	Total Cost
Private Insurance	5,088	18.7% ↑	$295	$1,265,905	4,784	17.4% ↓	$268	$1,059,899	199	17.2%	$258	$44,084	335	24.8% ↑	$278	$79,340
Medicaid	11,566	42.6% ↓	$217	$2,087,517	12,776	46.5% ↑	$282	$3,162,198	442	38.2% ↓	$234	$91,195	492	36.5% ↓	$301	$120,862
Medicare	2,356	8.7% ↑	$414	$829,977	2,100	7.6% ↓	$348	$646,394	35	3.0% ↓	$530	$14,303	78	5.8% ↓	$582	$37,241
Uninsured (Self-Pay)	7,748	28.5%	$209	$1,324,950	7,649	27.8% ↓	$260	$1,741,840	458	39.6% ↑	$282	$119,317	420	31.1% ↑	$288	$99,607
Other	414	1.5% ↑	$264	$96,004	194	0.7% ↓	$267	$43,536	23	2.0% ↑	$203	$3,861	24	1.8% ↑	$158	$3,634
Total:	27,172	100.0%	$248	$5,614,868	27,503	100.0%	$279	$6,668,503	1,157	100.0%	$263	$277,131	1,349	100.0%	$303	$344,071

	2012															
	White				Black				Hispanics				Other			
Payer	Count	%	Average Cost	Total Cost	Count	%	Average Cost	Total Cost	Count	%	Average Cost	Total Cost	Count	%	Average Cost	Total Cost
Private Insurance	4,982	18.7% ↑	$352	$1,491,491	4,919	16.4% ↓	$320	$1,321,686	237	15.2% ↓	$332	$68,729	395	24.6% ↑	$352	$113,801
Medicaid	11,311	42.5% ↓	$248	$2,355,467	14,284	47.7% ↑	$319	$4,057,541	666	42.6% ↓	$254	$147,127	578	36.0% ↓	$331	$158,272
Medicare	2,573	9.7% ↑	$415	$927,310	2,536	8.5% ↓	$398	$899,705	77	4.9% ↓	$306	$20,821	89	5.5% ↓	$413	$30,174
Uninsured (Self-Pay)	7,470	28.0%	$226	$1,388,596	8,079	27.0% ↓	$285	$2,018,367	560	35.8% ↑	$277	$135,764	513	32.0% ↑	$298	$119,037
Other	307	1.2% ↑	$395	$104,281	149	0.5% ↓	$350	$46,587	23	1.5% ↑	$279	$4,468	29	1.8% ↑	$466	$12,127
Total:	26,643	100.0%	$280	$6,269,330	29,967	100.0%	$317	$8,345,708	1,563	100.0%	$277	$376,909	1,604	100.0%	$334	$433,411

	2013															
	White				Black				Hispanics				Other			
Payer	Count	%	Average Cost	Total Cost	Count	%	Average Cost	Total Cost	Count	%	Average Cost	Total Cost	Count	%	Average Cost	Total Cost
Private Insurance	4,309	18.6% ↑	$383	$1,417,987	4,486	16.1% ↓	$353	$1,302,775	216	14.9% ↓	$322	$57,550	367	25.3% ↑	$402	$122,560
Medicaid	10,278	44.4% ↓	$329	$2,895,709	13,780	49.6% ↑	$341	$4,137,561	616	42.4% ↓	$274	$139,174	564	38.9% ↓	$296	$138,875
Medicare	2,289	9.9% ↑	$482	$964,884	2,230	8.0% ↓	$420	$841,123	52	3.6% ↓	$319	$14,666	87	6.0% ↑	$425	$31,421
Uninsured (Self-Pay)	5,973	25.8%	$261	$1,319,837	7,111	25.6% ↓	$311	$1,914,195	551	37.9% ↑	$310	$142,896	406	28.0% ↓	$308	$100,822
Other	317	1.4% ↑	$385	$108,225	186	0.7% ↓	$358	$58,793	17	1.2%	$722	$11,545	26	1.8% ↑	$242	$5,328
Total:	23,166	100.0%	$338	$6,709,003	27,793	100.0%	$342	$8,259,798	1,452	100.0%	$302	$365,831	1,450	100.0%	$333	$399,606

Note: ↑ indicates Chi-Square significantly higher than expected and ↓ indicates significantly lower than expected. Dental/Oral Health Related Conditions are defined as diagnoses of ICD-9-CM codes 520.0 through 529.9. Estimates from Maryland State Emergency Department Data (SEDD), 2013, Agency for Healthcare Research and Quality (AHRQ). Costs calculated using Cost-to-Charge Ratio Files for the State Inpatient Databases, 2013, Agency for Healthcare Research and Quality (AHRQ).
