# Supplementary figures and images for: Racial Disparities in Emergency Department Utilization for Dental/Oral Health-Related Conditions in Maryland
Source: Front Public Health. 2017 Jul 18;5:164. doi: 10.3389/fpubh.2017.00164 (PMC5515044; doi:10.3389/fpubh.2017.00164)

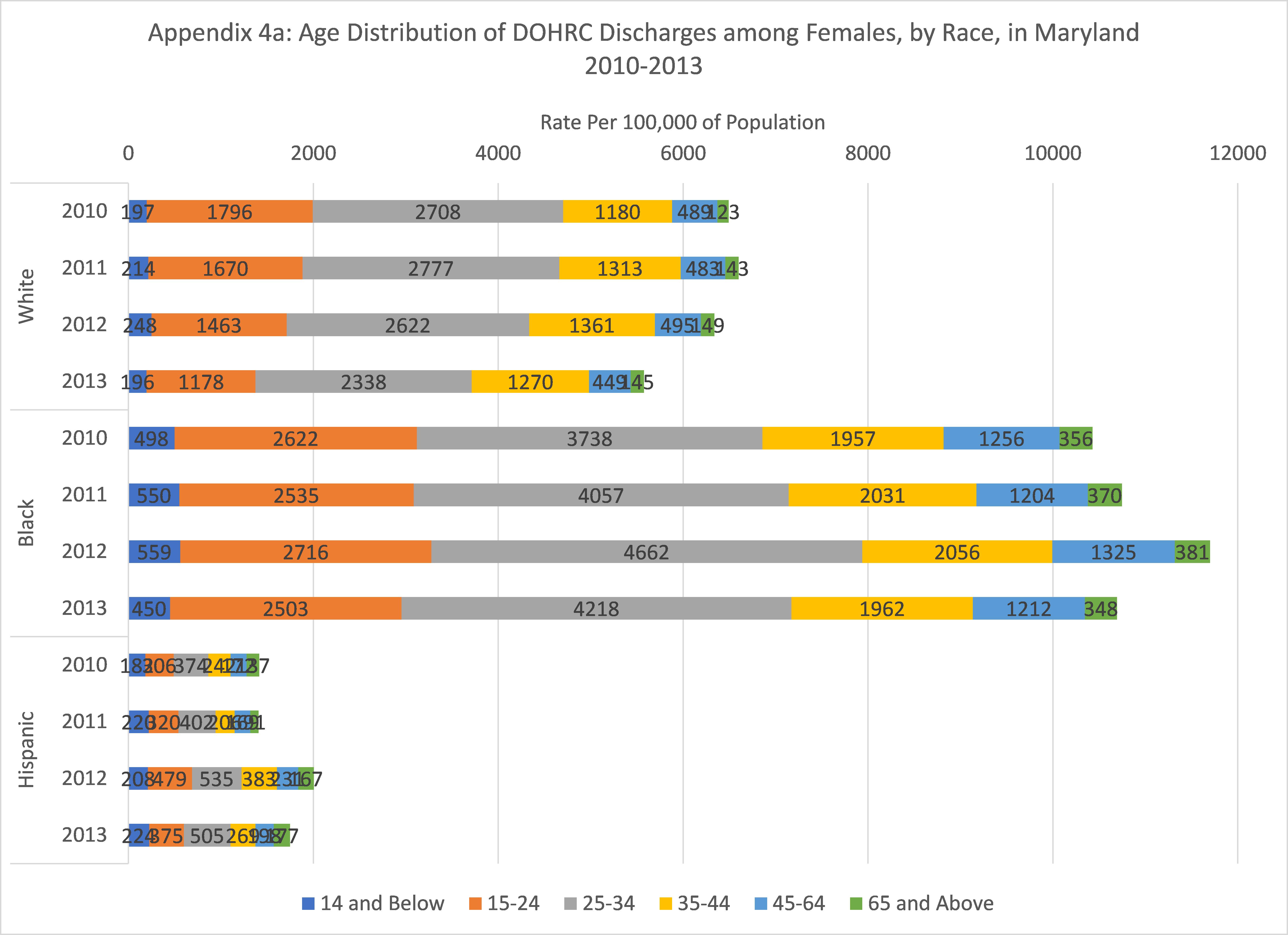

Supplement: Supplementary file 4 [file Image_1.JPEG]

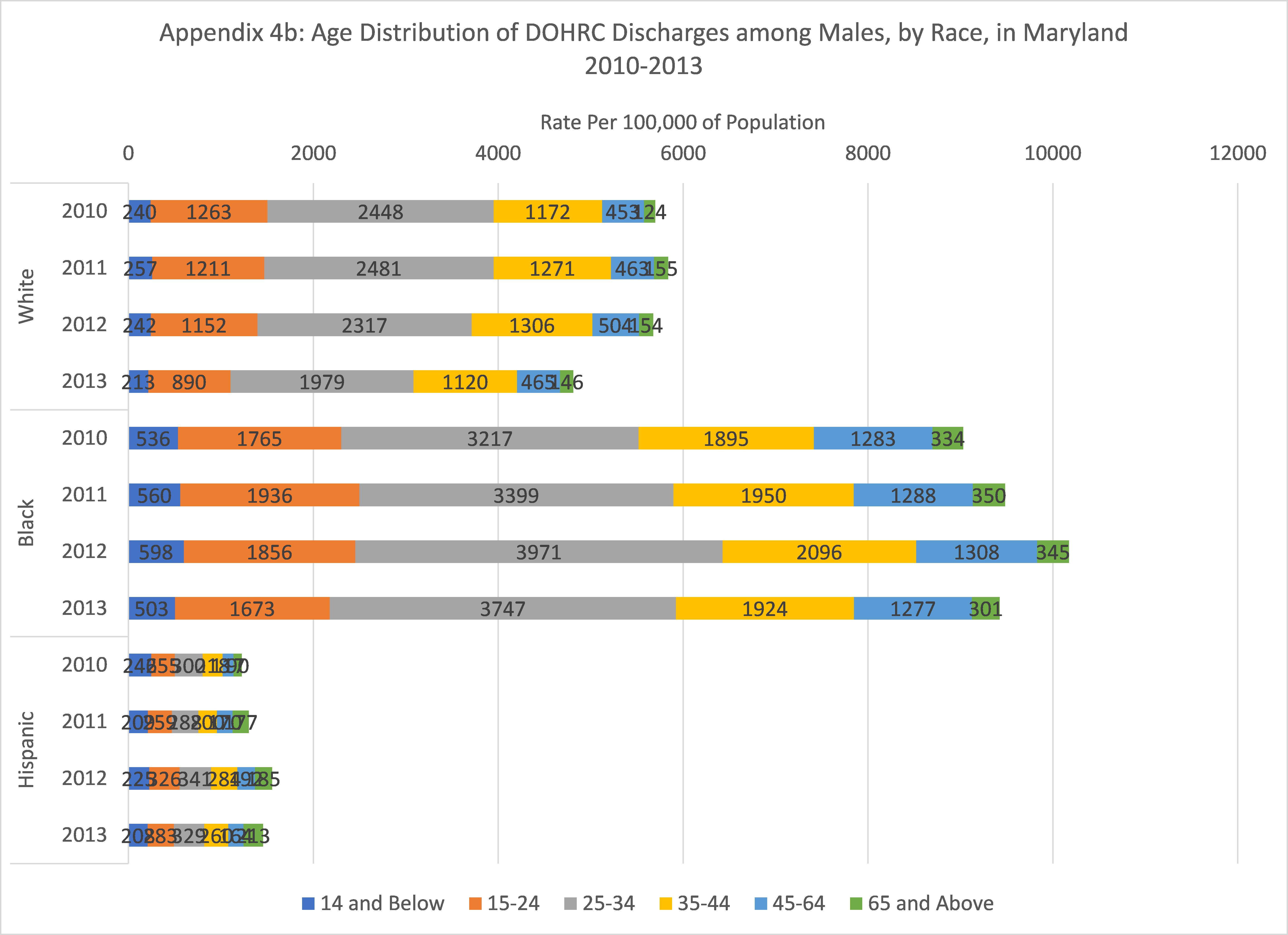

Supplement: Supplementary file 5 [file Image_2.JPEG]

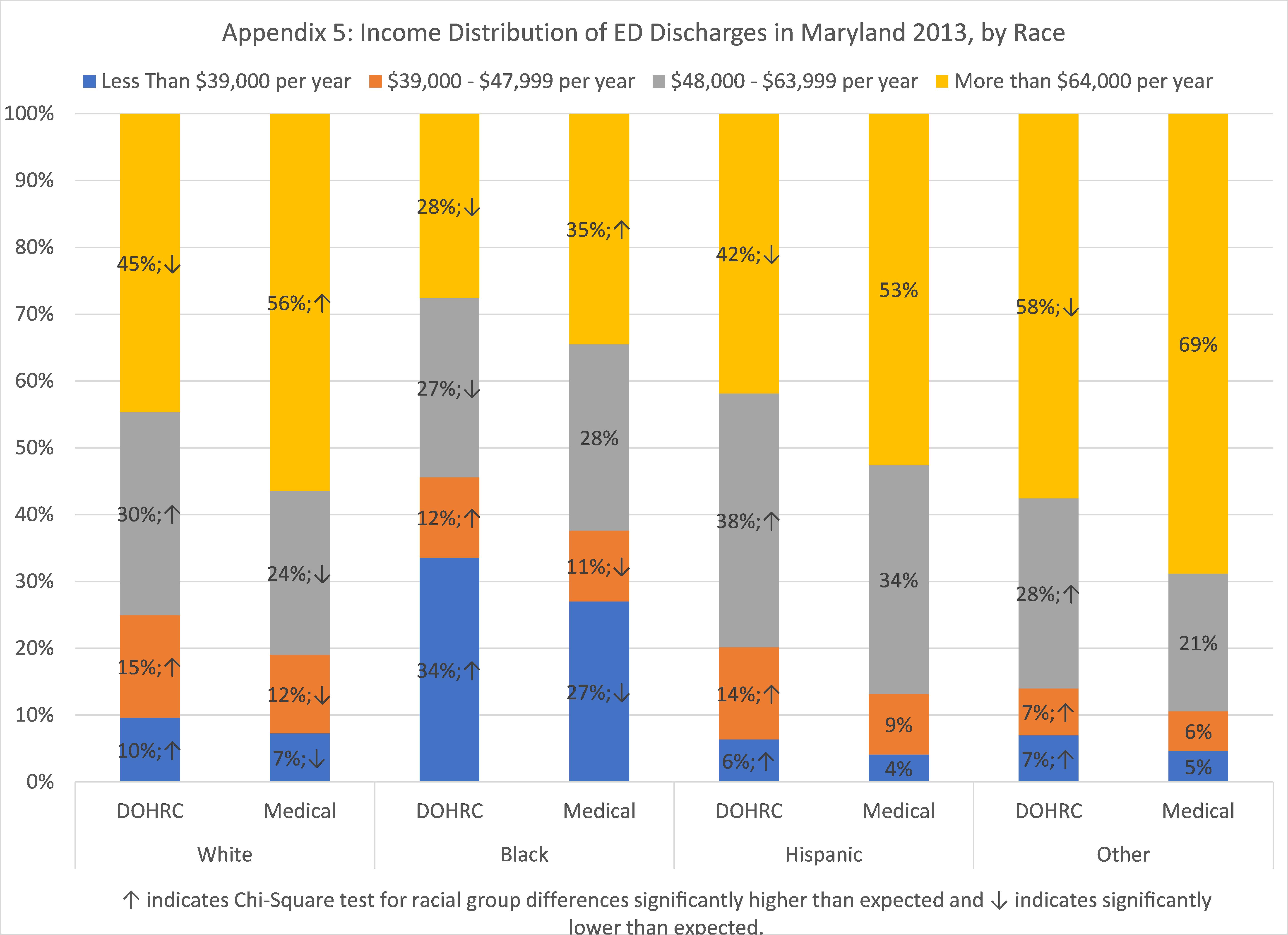

Supplement: Supplementary file 6 [file Image_3.JPEG]

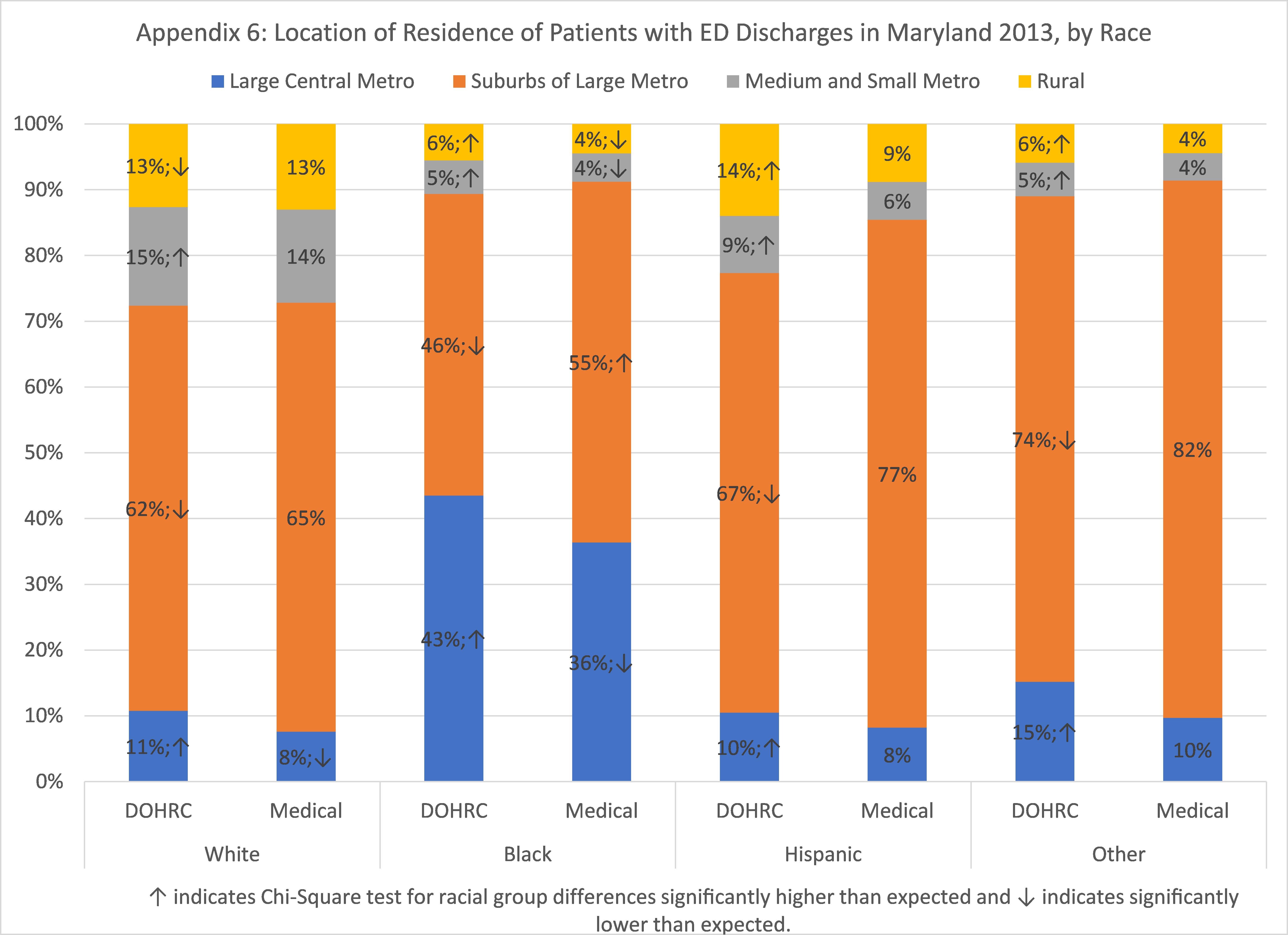

Supplement: Supplementary file 7 [file Image_4.JPEG]

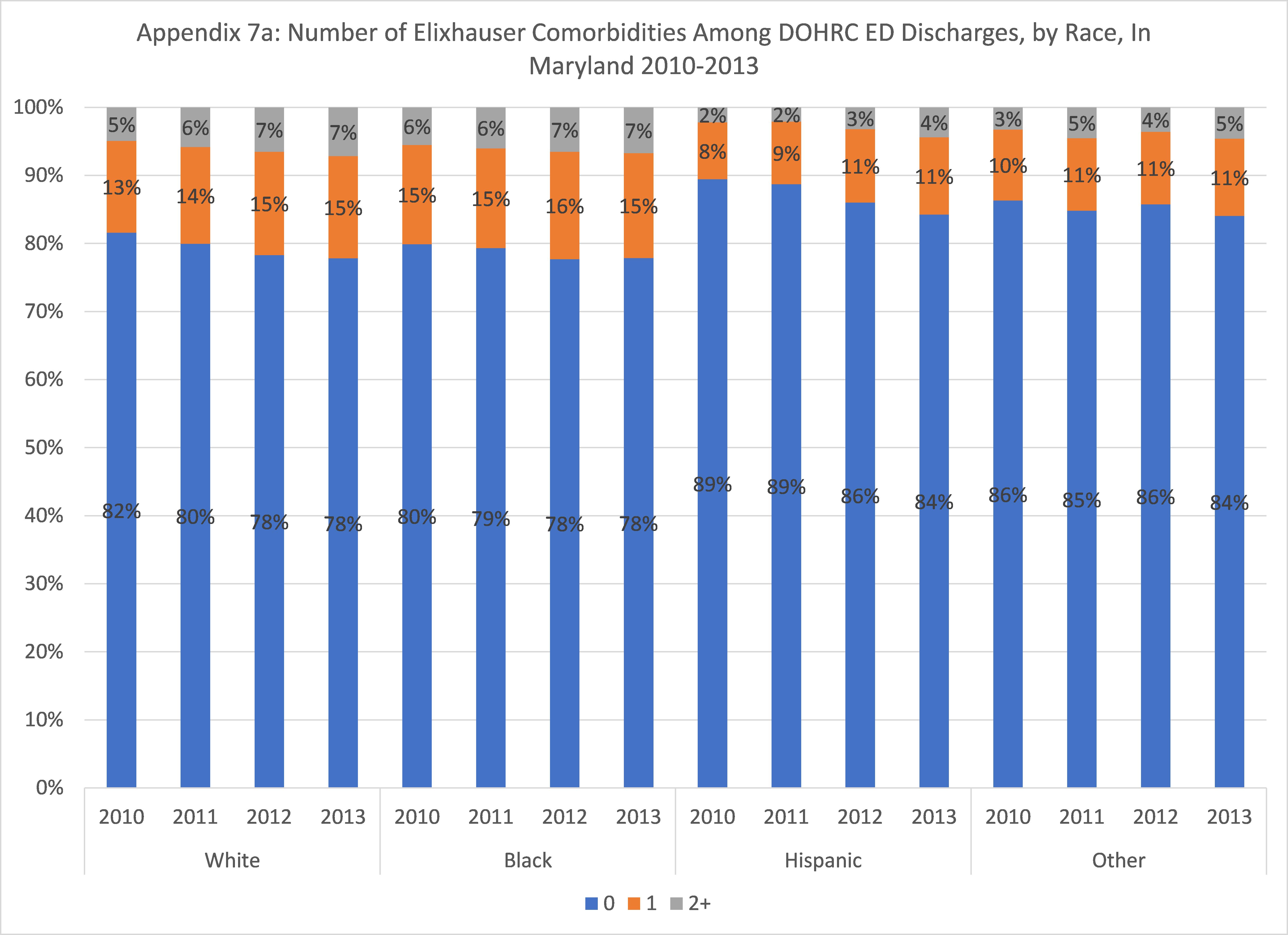

Supplement: Supplementary file 8 [file Image_5.JPEG]

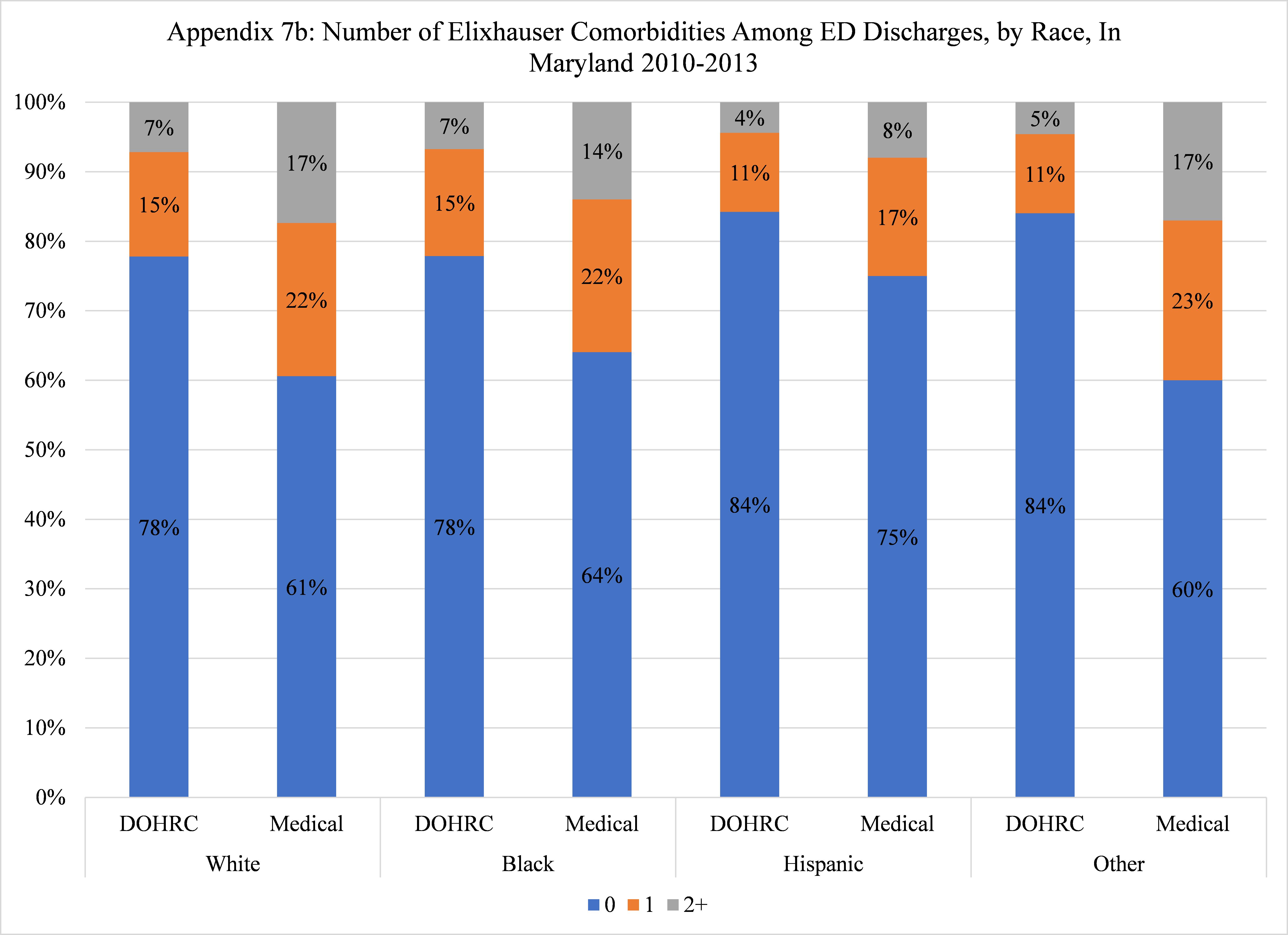

Supplement: Supplementary file 9 [file Image_6.JPEG]

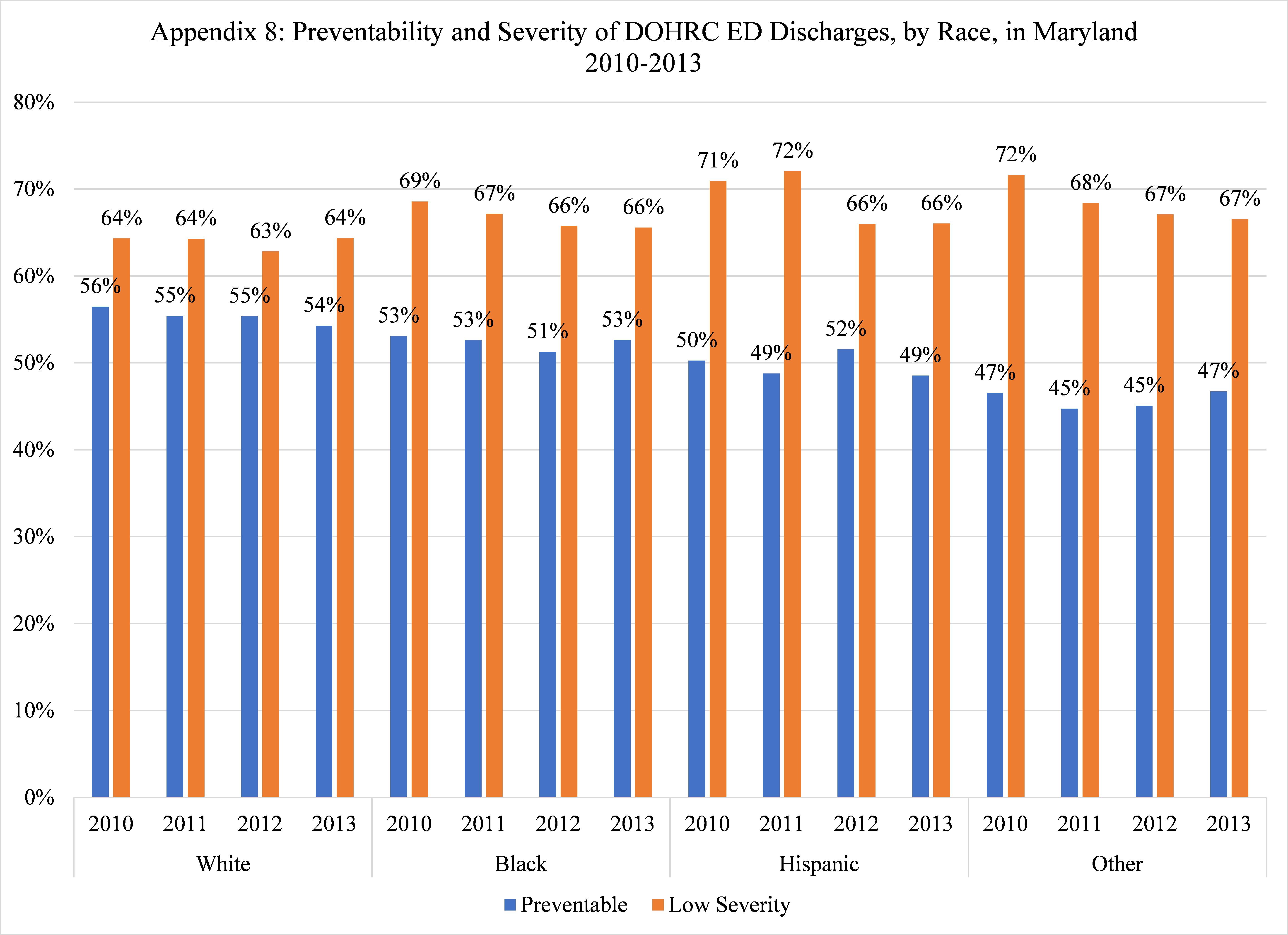

Supplement: Supplementary file 10 [file Image_7.JPEG]
